# RBM39 Contributes to MGMT Maintenance in Response to Temozolomide-Induced DNA Damage

**DOI:** 10.3390/cancers17223604

**Published:** 2025-11-08

**Authors:** Vahid Khalaj, Jack T. Adams, Solmaz AghaAmiri, Servando Hernandez Vargas, Tyler M. Bateman, Sukhen C. Ghosh, Majid Momeny, Ali Azhdarinia

**Affiliations:** The Brown Foundation Institute of Molecular Medicine, McGovern Medical School, The University of Texas Health Science Center at Houston, Houston, TX 77054, USA

**Keywords:** neuroendocrine neoplasms, MGMT, RBM39, temozolomide resistance, alkylating chemotherapy

## Abstract

**Simple Summary:**

O6-Methylguanine-DNA methyltransferase (MGMT) is a key DNA repair enzyme that protects genomic stability by removing alkyl adducts from the O6 position of guanine. Available data indicate that the elevated MGMT expression in glioblastoma and neuroendocrine neoplasms is associated with resistance to alkylating agents such as temozolomide (TMZ). In this study, we investigated the effect of RBM39 on MGMT expression, based on preliminary observations suggesting a potential connection between these two proteins. Pharmacological or knockdown depletion of RBM39 led to a significant reduction in MGMT protein levels and increased sensitivity of cancer cells to TMZ. Moreover, the combination of Indisulam, a pharmacological degrader of RBM39, with the MGMT inhibitor O6-benzylguanine (O6-BG) resulted in enhanced MGMT depletion. These findings suggest that co-targeting RBM39 and MGMT may represent a promising strategy to overcome chemoresistance in tumors with high MGMT activity.

**Abstract:**

Resistance to alkylating chemotherapeutic agents such as temozolomide (TMZ) is a significant challenge in treating tumors with high MGMT expression, including MGMT-positive glioblastoma and neuroendocrine neoplasms. In this study, we investigated the effect of RNA-binding motif protein 39 (RBM39) downregulation on MGMT protein levels, based on prior observations suggesting an association between these two proteins. Pharmacological depletion or siRNA-mediated knockdown of RBM39 led to a marked reduction in MGMT protein levels in MGMT-expressing cancer cells. We further showed that dual targeting of RBM39 (using indisulam) and MGMT (with O6-benzylguanine) synergistically enhanced MGMT depletion. Functionally, combined indisulam and TMZ treatment significantly increased apoptosis and decreased clonogenic growth in neuroendocrine tumor cells. These findings identify MGMT as a downstream target of RBM39 in MGMT-expressing cancer cells and highlight the therapeutic potential of co-targeting RBM39 and MGMT to overcome resistance to alkylating chemotherapy.

## 1. Introduction

O6-Methylguanine-DNA methyltransferase (MGMT) is a highly conserved 21 kDa nuclear DNA repair enzyme encoded by the MGMT gene, spanning 303,743 nucleotides on chromosome 10q26 [1]. This methyltransferase catalyzes the single-step transfer of an alkyl group from the O6 position of guanine to an active cysteine (Cys145) within the enzyme pocket. It directly reverses alkyl lesions in DNA without intermediary steps or generation of DNA strand breaks [2,3]. MGMT functions as a suicide enzyme, as it undergoes proteasomal degradation immediately after removing the alkyl group. Then, new protein synthesis and nuclear recruitment are necessary for MGMT recovery [4,5,6].

Targeting MGMT in tumors with elevated levels of this protein is essential, as its overexpression plays a significant role in resistance to the DNA alkylating agents such as temozolomide (TMZ) [6,7,8,9,10]. TMZ, the current standard chemotherapy for glioblastoma, induces DNA damage by methylating guanine and adenine bases. Approximately 70% of adducts occur at the N7 position of guanine (m7G) and 9% at the N3 position of adenine (m3A), and these are primarily repaired by base-excision repair (BER). However, the critical cytotoxic lesion is O6-methylguanine (~5% of total methylation events), which preferentially pairs with thymidine during replication. This mispairing promotes mismatch repair (MMR) cycles and generates lethal double-strand breaks that induce apoptosis [7,8]. In this context, the primary resistance to TMZ is directly linked to high MGMT expression [9]. The cellular level of MGMT is shown to be affected by the methylation status of its promoter. MGMT promoter methylation reduces the intracellular level of MGMT and is correlated with improved progression-free and overall survival in glioblastoma patients treated with alkylating agents [10,11,12,13,14]. Although the rate of methylation varies across neuroendocrine neoplasms, published data indicate that the promoter methylation predicts better outcomes to TMZ-based chemotherapy in patients with neuroendocrine tumors [15,16,17,18,19,20,21]. These clinical data highlight the key role of MGMT in mediating resistance to TMZ across various tumor types.

To overcome MGMT-mediated resistance, pharmacological inactivation using O6-benzylguanine (O6-BG) and O6-[4-bromothenyl]-guanine (O6-BTG, Lomeguatrib) has been explored as a therapeutic strategy. These pseudo-substrates mimic O6-methylguanine, react with MGMT similarly to alkylated DNA, and potentiate the cytotoxicity of alkylating agents by inhibiting MGMT-mediated DNA repair, but do not exhibit antitumor efficacy on their own [22,23]. Although these compounds have been tested in clinical trials, their use is limited due to hematological toxicity concerns [24,25]. In this regard, the development of safer and less toxic strategies for MGMT depletion is a priority.

While MGMT was traditionally seen as a standalone protein, recent studies have revealed a complex regulatory network that affects MGMT activity at transcriptional and post-translational levels [26,27,28,29]. In this sense, investigating the MGMT interactome is crucial for uncovering regulatory mechanisms that could reveal new strategies for targeting MGMT.

In our previous work, we validated the use of SNAP-Capture magnetic beads, which are surface-coupled with benzylguanine, for capturing an MGMT-GFP fusion protein [30]. Accordingly, we hypothesized that this SNAP-capture-based pulldown approach would selectively isolate the pool of MGMT proteins that remain functionally active and resistant to proteasomal degradation following TMZ treatment, while excluding MGMT that is inactivated through covalent DNA binding. Using this strategy, we performed a pulldown assay in TMZ-treated IMR-32 cells. This identified multiple nuclear and non-nuclear proteins associated with MGMT, including enriched RNA-binding protein 39 (RBM39) (unpublished data). Considering MGMT as a DNA damage response (DDR) gene, and based on prior evidence that RBM39 regulates DNA repair enzymes, we further investigated the impact of RBM39 depletion on MGMT levels [31].

In the present communication, we targeted RBM39 through both pharmacological inhibition with indisulam and siRNA-mediated knockdown to evaluate whether RBM39 downregulation affects MGMT expression. To the best of our knowledge, this is the first study to indicate a potential connection between RBM39 and MGMT. Our findings demonstrate the role of RBM39 in modulating MGMT expression levels and introduce RBM39 as a potential candidate to circumvent alkylating agent resistance.

## 2. Materials and Methods

### 2.1. Cell Lines, Chemicals, and Antibodies

Cell lines IMR-32 (human neuroblastoma), MCF-7 (human breast adenocarcinoma), and QGP-1 (human pancreatic neuroendocrine tumor) were selected to investigate the effects of RBM39 downregulation across distinct tissue origins. These cell lines were selected to determine whether the results of RBM39 depletion could be reproduced across diverse cancer types and tissue backgrounds, and because they exhibit robust MGMT expression. The cells were cultured in DMEM or RPMI medium supplemented with 10% bovine serum albumin and maintained at 37 °C with 95% humidity and 5% CO_2_ atmosphere. Line-specific media were as follows: IMR-32: RPMI-1640 + 10% FBS; MCF-7: DMEM + 10% FBS and QGP-1: RPMI-1640 + 10% FBS. O6-Benzylguanine (MGMT inhibitor) and indisulam (RBM39 degradation inducer) were obtained from AdooQ^®^ Bioscience (Irvine, CA, USA) and dissolved in dimethyl sulfoxide (DMSO) at a concentration of 20 mM. Working concentrations for indisulam were chosen per line based on dose–response viability and were consistent with prior literature [32,33]. Temozolomide was acquired from TCI (Portland, OR, USA) and prepared as a 100 mM stock solution in DMSO for subsequent use. Rabbit monoclonal anti-MGMT antibody (EPR4397), rabbit monoclonal anti-Cleaved poly (ADP-ribose) polymerase (PARP) [E51] antibody, and mouse monoclonal anti-actin antibody (EPR16769) were obtained from Abcam (Waltham, MA, USA). RBM39 monoclonal antibody was purchased from Proteintech (67420-1-Ig). IRDye^®^ 800CW goat anti-rabbit IgG secondary antibody and IRDye^®^ 680RD goat anti-mouse IgG secondary antibody were purchased from LI-COR (Lincoln, NE, USA).

### 2.2. Clonogenic Assay

Long-term cytotoxicity was assessed using a clonogenic assay of QGP-1 cells in response to TMZ, indisulam, and the combination of the two compounds compared to vehicle-treated control. QGP-1 cells were seeded at a cell density of 1000 cells per well in 6-well plates and allowed to adhere overnight. Cells were then treated with TMZ, indisulam, combination, or vehicle control for 72 h. Following treatment, the media was subsequently replaced with drug-free media, and the cells were allowed to grow for a total of 14 days post-seeding. After the growth period, the cells were washed with PBS, fixed with ice-cold methanol for 30 min, and then stained with crystal violet solution (0.5 g crystal violet, 25 mL methanol, and 75 mL MiliQ H20) for 1 hr. The colonies were then manually counted when they reached a size of more than 50 cells. Plating efficiency was calculated by taking the number of colonies formed in the control treatment divided by the number of seeded cells. Treatment groups were then analyzed to a normalized surviving fraction by taking the number of colonies formed after treatment, divided by the number of cells seeded, multiplied by the plating efficiency.

### 2.3. Western Blot Analysis

Changes in the expression levels of MGMT and RBM39 proteins were assessed in response to various treatments or siRNA-mediated knockdown of RBM39 using Western blotting. Upon drug treatments or knockdown experiments, total cell lysates were separated using SurePAGE™ Precast Gels (GenScript, Piscataway, NJ, USA) and transferred to a nitrocellulose membrane. The membranes were blocked with Intercept Blocking Buffer (LI-COR) and subsequently incubated with specific primary antibodies (RBM39, 1:2000; MGMT, 1:5000; ACTIN, 1:10,000), followed by IR-labeled secondary antibodies (1:5000). Immunoreactive bands were visualized using the LI-COR Odyssey Western blot imager. Additionally, the induction of cleaved PARP, a marker of apoptosis, was analyzed with the same Western blotting technique. Statistical analysis of Western blot densitometry data was performed using one-way ANOVA in Prism version 10 (GraphPad Software). Band intensities were normalized to the corresponding loading control before analysis. All quantitative measurements were obtained from at least three independent (triplicate) blots. Data are presented as mean ± SEM, with statistical significance set at *p* < 0.05.

### 2.4. siRNA Knockdown

Three predesigned siRNA duplexes targeting human RBM39 (siRNA A, B, and C; Cat. Nos. SR306375A, SR306375B, and SR306375C) were purchased from OriGene Technologies (Rockville, MD, USA). IMR-32 cells were transfected individually with each siRNA using the manufacturer’s protocol, and knockdown efficiency was evaluated by Western blot analysis. Among the three, siRNA A (rCrCrUrCrUrArGrCrArArUrArGrGrArUrUrArArCrUrGrGCC) achieved the most efficient and consistent downregulation of RBM39 and was selected for all subsequent experiments. To optimize transfection conditions, cells were treated with siRNA A at various concentrations (5, 10, 20, 50, 100 nM) and harvested at different time points (12, 24, 48, and 72 h) to assess knockdown efficiency. A concentration of 50 nM was identified as optimal, providing robust RBM39 knockdown without detectable cytotoxicity, and was used for all further studies.

Cells were seeded overnight in 6-well plates (IMR-32: 5 × 10^5^ cells/well) and transfected with siRNA A or a universal scrambled negative control siRNA duplex (SR30004, OriGene). Since the most significant reduction in MGMT expression was observed 72 h after indisulam treatment, this time point was also chosen for harvesting cells and analyzing the effect of RBM39 knockdown on MGMT levels.

## 3. Results

### 3.1. Snap-Capture Magnetic Beads Enable Affinity-Based Isolation of Active MGMT Complexes and Reveal Nuclear Interactors Following Temozolomide Treatment

Building upon our previous work, which demonstrated the specific immobilization of MGMT by Snap-Capture magnetic beads, we hypothesized that the active MGMT repair enzyme, in complex with its associated proteins, could be isolated using this affinity-based approach before its deactivation by alkylated DNA during the repair process. A pulldown assay was performed on IMR-32 cells treated with temozolomide (60 µM), using SNAP-capture beads for MGMT immobilization, following a method similar to that described in our previous publication [30]. While a comprehensive analysis of all MGMT-associated proteins was performed, only findings directly relevant to the current study are presented here. In this targeted analysis, several proteins were found to co-capture with MGMT under the tested condition, with a particular focus on nuclear proteins exhibiting the highest abundance post-treatment. Of interest, RBM39 was enriched together with BET family members following TMZ treatment. As it has been previously demonstrated that BET inhibition reduces MGMT expression [28], we focused our attention on investigating the role of RBM39 in MGMT expression.

### 3.2. Indisulam Triggers Early RBM39 Depletion and Subsequent MGMT Suppression

**Figure 1:**
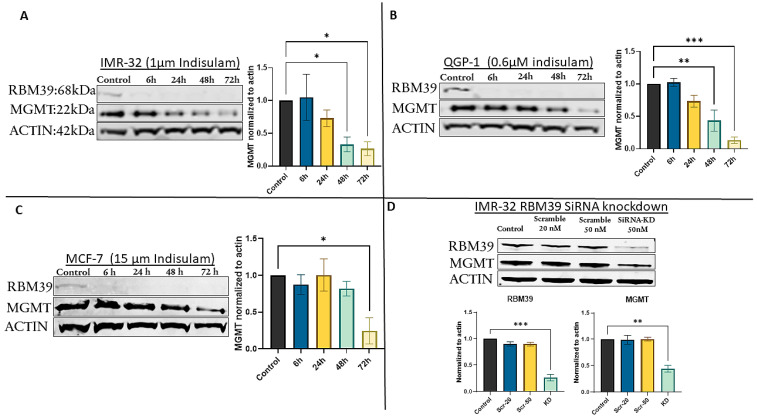
Indisulam treatment induces RBM39 degradation and downregulation of MGMT in multiple cancer cell lines. (**A**) IMR-32, (**B**) QGP-1, and (**C**) MCF-7 cells were treated with indisulam for the indicated times. (**D**) RBM39 siRNA knockdown in IMR-32 cells recapitulates MGMT suppression. β-actin was used as a loading control. Bar graphs represent densitometric quantification of RBM39 and MGMT band intensities normalized to β-actin and expressed relative to the control condition (mean ± SD, *n* = 3). Statistical significance was determined by one-way ANOVA; *p* < 0.05 (*), *p* < 0.005 (**), *p* < 0.0005 (***). The uncropped blots are shown in Figure S1 and Table S1.

We initially performed our proof-of-concept study in the IMR-32 cell line. IMR-32 cells were treated with efficacious doses of indisulam (1 µM) for 72 h [33], and then changes in the expression levels of RBM39 and MGMT were assessed using Western blot analysis. As illustrated in Figure 1A, in the early hours of indisulam treatment, a significant reduction of RBM39 was observed, with a marked decrease of the RBM39 band as early as 6 h post-treatment. This reduction was sustained throughout the experiment, indicating the effectiveness of indisulam in targeting and suppressing RBM39 expression. Following the indisulam-induced, DCAF15-dependent RBM39 degradation, we observed a gradual decrease in MGMT expression [34,35]. This reduction became most prominent on day three of the experiment, indicating a potential correlation between RBM39 suppression and decreased MGMT levels (Figure 1A). To further validate these findings, the effect of RBM39 downregulation was investigated in two additional cell lines, QGP-1 and MCF-7. Efficacious doses of indisulam were identified (0.6 µM for QGP-1 and 15µM for MCF-7) and used for treatment. These cell lines were chosen as they are more robust and tractable MGMT-positive models [15,36]. The results demonstrated a similar reduction pattern for MGMT expression, with the highest level of suppression observed at 72 h post-treatment (Figure 1B,C). In MCF-7 cells, the reduction in MGMT was comparatively modest, which may reflect the high basal expression of MGMT in this cell line. Such elevated baseline levels likely confer greater protein stability and may delay or limit the extent of MGMT reduction following RBM39 degradation.

### 3.3. siRNA-Mediated RBM39 Knockdown Confirms MGMT Downregulation Is a Direct Consequence of RBM39 Loss

To confirm that MGMT downregulation was not an off-target effect of indisulam, we used siRNA A (SR306375A, OriGene Technologies) for specific knockdown of RBM39 without pharmacological intervention. In IMR-32 cells, the most responsive to knockdown, RBM39 knockdown led to a significant reduction in both RBM39 and MGMT, confirming that MGMT depletion results from RBM39 loss rather than an off-target effect of indisulam (Figure 1D).

### 3.4. MGMT Inhibition Does Not Affect RBM39 Expression

**Figure 2:**
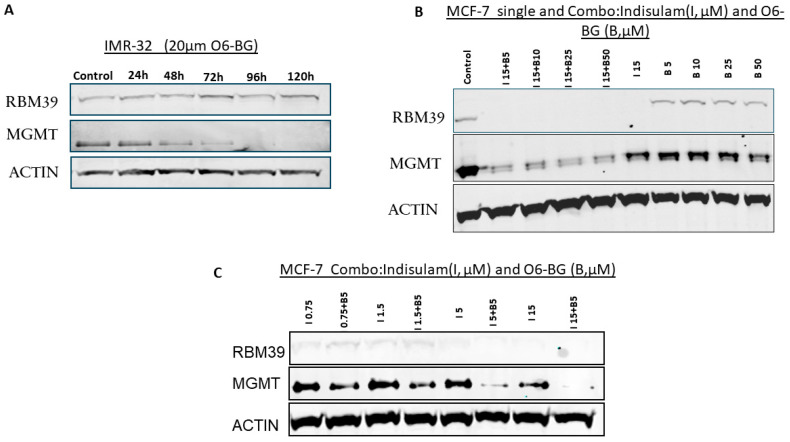
Effects of O6-BG and combined treatment with indisulam on RBM39 and MGMT expression. (**A**) IMR-32 cells treated with 20 μM O6-BG for 24–120 h show consistent RBM39 levels but progressive MGMT suppression. (**B**) MCF-7 cells treated with indisulam (I, 15 μM), O6-BG (B, 5–50 μM), or I + B demonstrate RBM39 degradation by indisulam and significant MGMT suppression by O6-BG. Combination treatment further enhances MGMT downregulation. (**C**) Dose–response studies in MCF-7 cells (indisulam 0.75–15 μM ± O6-BG 5 μM) to determine the efficient minimum dose for synergistic MGMT suppression under combination therapy. β-actin was used as a loading control. The uncropped blots are shown in Figure S2 and Table S1.

The effect of MGMT depletion on RBM39 expression was investigated using the MGMT-specific inhibitor O6-BG. This was done to determine whether RBM39 expression is influenced by the inactivation of MGMT using O6-BG. The treatment of IMR-32 cells with O6-BG did not affect RBM39 levels even after 5 days, demonstrating that RBM39 expression remains unchanged despite MGMT downregulation (Figure 2A). These results indicate that the relationship between RBM39 and MGMT is not reciprocal and suggest that RBM39 may function as a master regulator of MGMT expression, similar to its established role in controlling other key DNA repair machinery [31].

### 3.5. Combination Treatment with Indisulam and O6-BG Synergistically Depletes MGMT

To exploit the potential synergistic effects of targeting both RBM39 and MGMT and achieve maximum MGMT depletion, we used a combination treatment approach with different concentrations of indisulam and O6-BG. MCF-7 cells were exposed to a range of indisulam concentrations for 72 h. O6-BG was then introduced at the 48 h time point, allowing for a 24 h treatment period with O6-BG before the experiment was completed. Figure 2B illustrates that indisulam (15 µM) depletes RBM39 and partially reduces MGMT levels, whereas O6-BG alone strongly suppresses MGMT without affecting RBM39. The combination treatment sustains RBM39 degradation and enhances MGMT suppression compared with single agents. To determine the synergistic minimum dose of the inhibitors while maintaining maximum efficacy, we also tested a dose-reduction strategy. Our results demonstrated that the combination of 5 μM indisulam and 5 μM O6-BG effectively reduced MGMT expression. Importantly, these concentrations represent the minimum effective doses that achieve efficient MGMT degradation in this high MGMT-expressing cell line, while avoiding the higher toxicity associated with single-agent high-dose treatments (Figure 2C).

### 3.6. Co-Treatment with Indisulam and TMZ Enhances Apoptosis and Inhibits Clonogenic Growth

**Figure 3:**
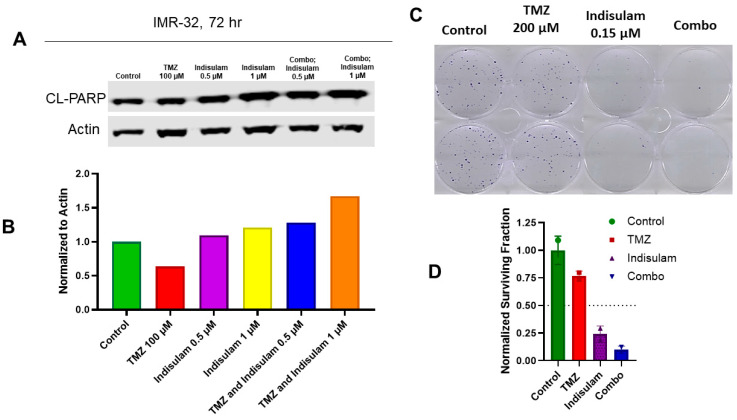
Indisulam enhances TMZ-induced apoptosis and reduces clonogenic survival. (**A**) Western blot analysis of IMR-32 cells treated with TMZ (100 μM), indisulam (0.5 or 1 μM), or combinations for 72 h. Cleaved PARP (CL-PARP) was used as a marker of apoptosis; β-actin was used as a loading control. (**B**) Quantification of CL-PARP expression normalized to actin. (**C**) Clonogenic assay of QGP-1 cells treated with control, TMZ (200 μM), indisulam (0.15 μM), or combination for 72 h, followed by growth in drug-free media for 14 d. (**D**) Colonies (>50 cells/colony) were stained with 0.5% crystal violet, counted, and normalized to the control to calculate the surviving fraction. The uncropped blots are shown in Figure S3 and Table S1.

Co-treatments of indisulam and TMZ were designed to determine cytotoxic effects in the MGMT-positive cell line, IMR-32. Since MGMT reduction following treatment with indisulam was strongest at 72 h, we hypothesized that reducing MGMT via indisulam could enhance the induction of apoptosis and attenuate clonogenic survival with TMZ treatment. IMR-32 cells were treated with 0.5 or 1 µM indisulam, 100 µM TMZ, or the combination of indisulam at both doses and TMZ. After 72 h, a remarkable increase in a marker of apoptosis, cleaved PARP, was observed in the combination group compared to either single agent (Figure 3A,B). Furthermore, to understand the long-term effect of inhibiting cancer cell growth, a clonogenic assay was performed. Following 72 h treatments with 0.15 µM indisulam, 200 µM TMZ, and their combination in QGP-1 cells, the drug combination inhibited the growth of clones to a much greater extent than the single agents alone (Figure 3C,D).

## 4. Discussion

MGMT repairs cytotoxic lesions caused by TMZ-mediated alkylation of DNA by removing the alkyl group through a suicidal transfer reaction, which irreversibly depletes MGMT protein levels in tumor cells [37]. In this study, we speculated that in response to MGMT depletion, cells may attempt to compensate for the loss of DNA repair capacity by recruiting additional MGMT, along with potential interacting partners, to restore the repair machinery. Several proteins were found to co-capture with MGMT after administering TMZ and employing SNAP-capture beads for MGMT immobilization. Our focus was on nuclear proteins, and we identified multiple proteins with high abundance post-treatment (Personal observation, 2025). As RBM39 was among the proteins with the highest abundance in this analysis, we focused on investigating its role in MGMT expression.

Recent studies have demonstrated that RBM39 plays a crucial role in regulating genes involved in the DNA damage response. For instance, global transcriptomic analyses reveal that RBM39 regulates both alternative splicing and transcription of genes linked to cell cycle progression and cellular response to DNA damage, highlighting its involvement in DDR pathways. This regulatory effect provides a mechanistic rationale for exploring how RBM39 downregulation might influence MGMT expression in the context of DNA-damaging agents such as TMZ [31,38].

Indisulam targets RBM39 by promoting the formation of a ternary complex with the E3 ubiquitin ligase receptor DCAF15 and DDB1, leading to proteasomal degradation of RBM39 [35,36]. We used this chemical inhibitor to follow the fate of MGMT. Our results demonstrated that upon RBM39 downregulation, MGMT levels dropped, while inhibition of MGMT using O6-BG did not alter RBM39 expression levels. In parallel, the siRNA-mediated knockdown of RBM39 resulted in downregulation of MGMT, which may indicate an on-target regulatory function of RBM39 rather than an off-target artifact. Although the published links between RBM39 and MGMT are still emerging, our results suggest that RBM39 may play a master regulatory role over MGMT. As RBM39 is a splicing regulator, we monitored the transcript size of MGMT and found no change following indisulam treatment. To the best of our knowledge, MGMT has not been identified as a direct splicing target of RBM39 in RNA-seq or proteomic analyses from neuroblastoma and leukemia models, which include large-scale RBM39 degrader transcriptomes cataloging numerous high-confidence splicing targets [34,39]. In this sense, suppression of MGMT following RBM39 degradation likely occurs through indirect mechanisms. Because RBM39 is a splicing factor essential for the expression of DNA repair genes such as BRCA1, BRCA2, ATM, and ATR [28], its loss can broadly disrupt the DNA repair network, which affects MGMT as a part of this machinery [40]. Additionally, RBM39 influences several oncogenic signaling pathways known to regulate MGMT transcription [26]. PI3K/AKT is one of these pathways, which has been shown to promote MGMT expression in multiple tumor types by activating transcriptional regulators such as Sp1 [26,41]. Silencing RBM39 has been shown to suppress PI3K/AKT activity in hepatocellular carcinoma (HCC) and acute myeloid leukemia (AML), leading to impaired cell proliferation and tumor growth [42,43]. Since Sp1 is a major transcriptional activator of MGMT and is regulated downstream of PI3K/AKT, the downregulation of MGMT following RBM39 depletion may partially result from reduced Sp1 activity due to diminished PI3K/AKT signaling. Moreover, the NF-κB pathway, another important transcriptional driver of MGMT, is also influenced by RBM39. RBM39 depletion has been shown to suppress NF-κB signaling [44], and inhibition of NF-κB is known to reduce MGMT transcription and sensitize glioma cells to TMZ [45]. Further to the above, it has been reported that RBM39 expression is broadly correlated with DNA methylation and DNA methyltransferase expression across various cancers, suggesting that RBM39 may influence the epigenetic landscape of tumor cells [46]. While MGMT was not particularly examined in that study, these findings raise the possibility that RBM39 depletion could change MGMT promoter methylation status as an additional mechanism contributing to the observed reduction in MGMT expression.

Beyond transcriptional regulation, RBM39 may also indirectly affect post-transcriptional mechanisms. For instance, miR-4261 has been reported to bind the 3′-UTR of MGMT and promote its translational repression [47]. Although our study did not examine the RBM39–miRNA axis, there is a possibility that RBM39 downregulation alters the availability or activity of MGMT-targeting miRNA(s), thereby affecting MGMT expression.

Collectively, our data suggest that RBM39 may indirectly regulate MGMT expression through transcriptional and post-transcriptional pathways. However, this study is limited by the lack of detailed mechanistic examinations, as it remains uncertain whether MGMT downregulation results from affected signaling pathways, changes in promoter accessibility, mRNA stability, or even broader epigenetic modifications. Additionally, our findings have not yet been validated in cancer models where temozolomide is a standard-of-care therapy, such as glioblastoma, which represents another limitation. While indisulam monotherapy has shown limited anti-tumor activity, albeit with acceptable toxicity [48], combining RBM39 degraders with MGMT inhibitors such as O6-BG could yield synergistic suppression of MGMT with reduced toxicity. This combination therapy may provide a strong rationale for future preclinical and clinical studies to overcome TMZ resistance in glioblastoma and neuroendocrine neoplasms.

## 5. Conclusions

Our findings demonstrate that RBM39 downregulation significantly decreases MGMT protein levels, likely through multiple transcriptional and post-transcriptional mechanisms. While the precise mechanisms remain to be fully elucidated, these findings provide a strong rationale for co-targeting RBM39 and MGMT to overcome temozolomide resistance in cancers with high MGMT expression, including glioblastoma and neuroendocrine neoplasms. Further preclinical and clinical studies are warranted to evaluate this combination as a promising therapeutic strategy.

## Data Availability

Data from this study are available upon reasonable request from the corresponding authors.

## Supplementary Materials

The following supporting information can be downloaded at: https://www.mdpi.com/article/10.3390/cancers17223604/s1, Figure S1: Uncropped Western blots corresponding to Figure 1; Figure S2: Uncropped Western blots corresponding to Figure 2; Figure S3: Uncropped Western blots corresponding to Figure 3; Table S1. Densitometric Analysis of Western Blot Bands Corresponding to Figure 1, Figure 2 and Figure 3.
