# Supplementary figures and images for: RBM39 Contributes to MGMT Maintenance in Response to Temozolomide-Induced DNA Damage
Source: Cancers (Basel). 2025 Nov 8;17(22):3604. doi: 10.3390/cancers17223604 (PMC12651708; doi:10.3390/cancers17223604)

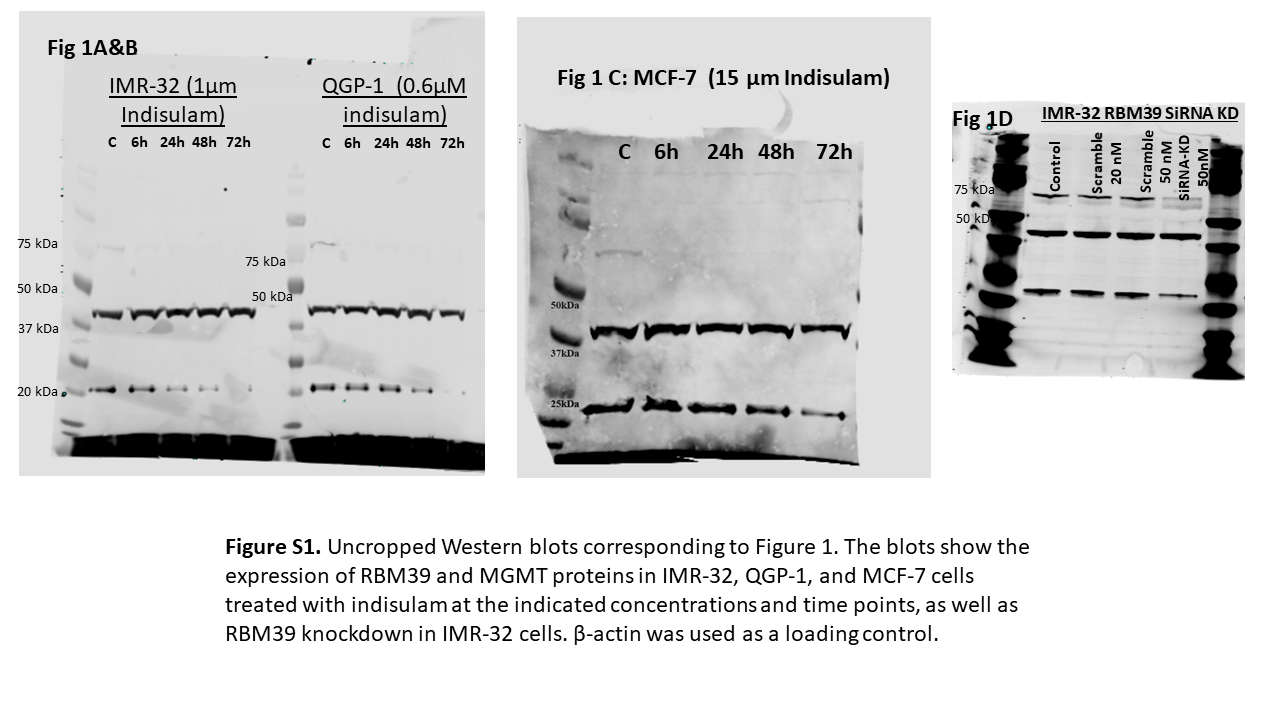

Supplement: Supplementary file 1 [file cancers-17-03604-s001.zip › Supplementary materials/Figure S1.png]

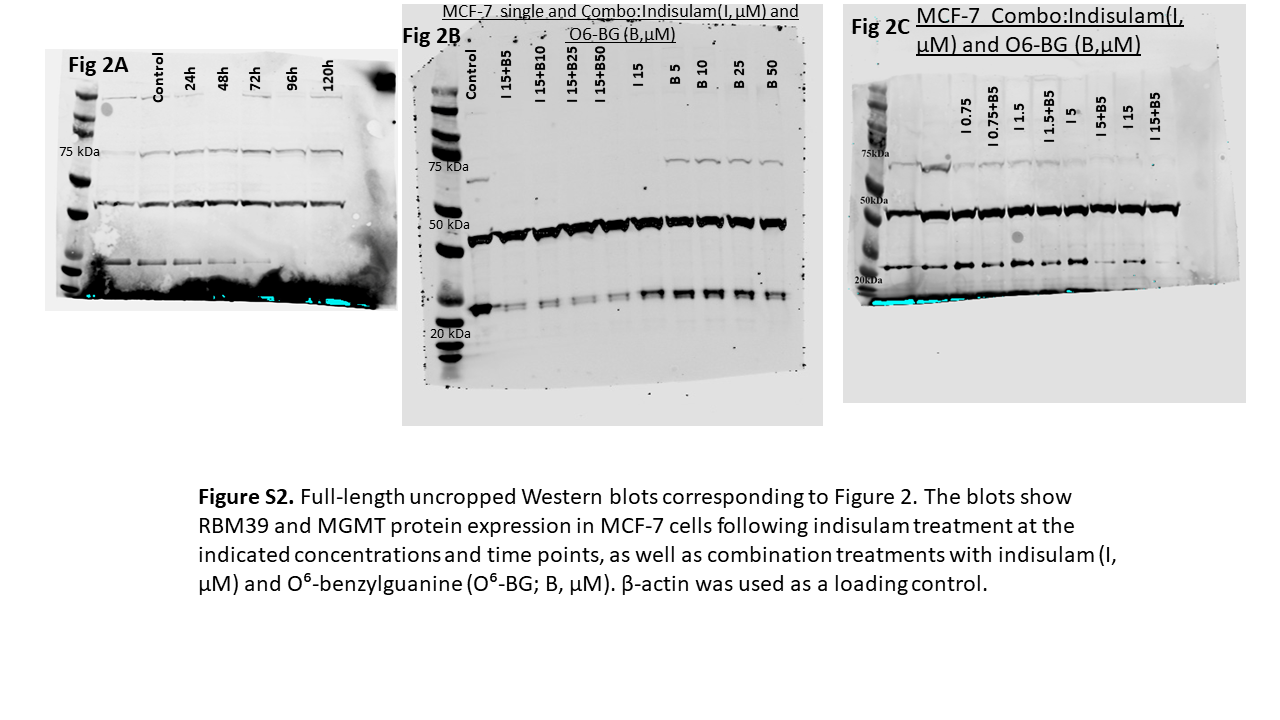

Supplement: Supplementary file 1 [file cancers-17-03604-s001.zip › Supplementary materials/Figure S2.png]

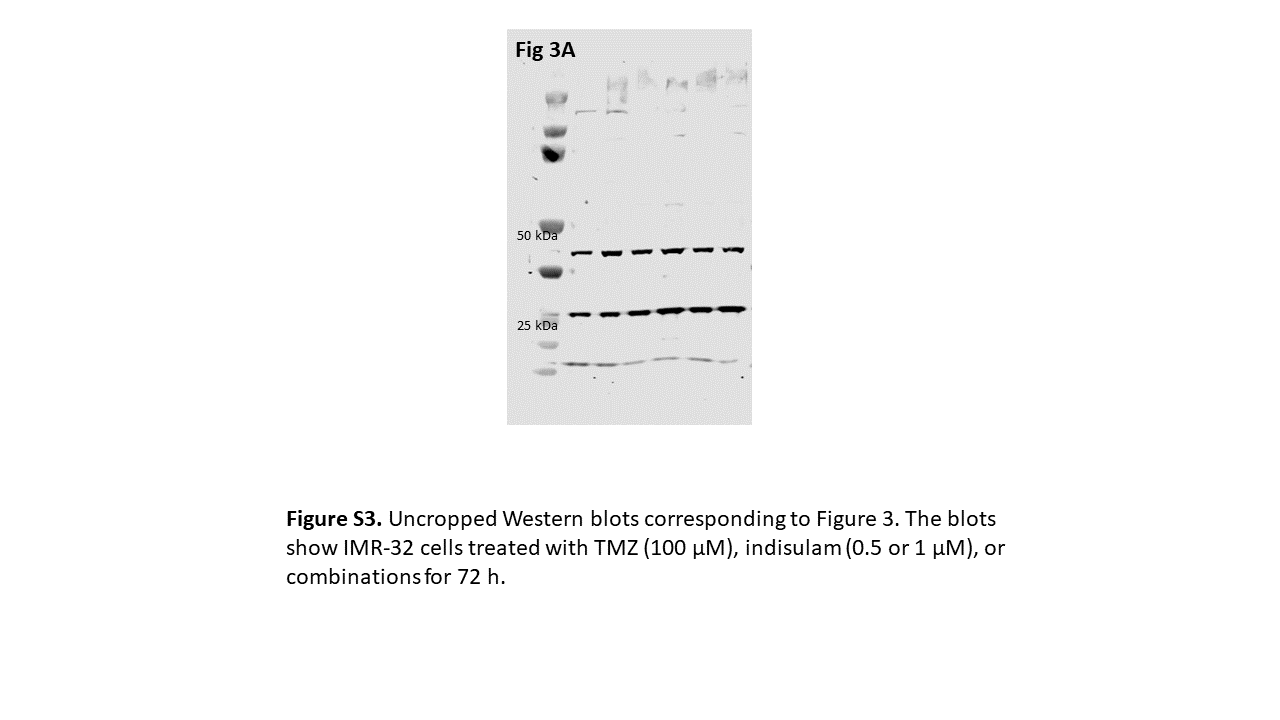

Supplement: Supplementary file 1 [file cancers-17-03604-s001.zip › Supplementary materials/Figure S3.png]
